# Crystal Engineering of Schiff Base Zn(II) and Cd(II) Homo- and Zn(II)M(II) (M = Mn or Cd) Heterometallic Coordination Polymers and Their Ability to Accommodate Solvent Guest Molecules

**DOI:** 10.3390/molecules26082317

**Published:** 2021-04-16

**Authors:** Olga Danilescu, Paulina N. Bourosh, Oleg Petuhov, Olga V. Kulikova, Ion Bulhac, Yurii M. Chumakov, Lilia Croitor

**Affiliations:** 1 Institute of Chemistry, Academy Str. 3, MD2028 Chisinau, Moldova; olgadanilescu@mail.ru (O.D.); bourosh.xray@gmail.com (P.N.B.); petuhov.chem@gmail.com (O.P.); ionbulhac@yahoo.com (I.B.); 2 Institute of Applied Physics, Academy Str. 5, MD2028 Chisinau, Moldova; olga.kulikova@ifa.md (O.V.K.); xray52@mail.ru (Y.M.C.); 3 Institute of Geology and Seismology, Gheorghe Asachi Str. 60/3, MD2028 Chisinau, Moldova

**Keywords:** coordination polymer, Zn(II), Cd(II), Schiff base, crystal structure, degassing, adsorption–desorption property, photoluminescence

## Abstract

Based on solvothermal synthesis, self-assembly of the heptadentate 2,6-diacetylpyridine bis(nicotinoylhydrazone) Schiff base ligand (H_2_L) and Zn(II) and/or Cd(II) salts has led to the formation of three homometallic [CdL]_n_ (**1**), {[CdL]∙0.5dmf∙H_2_O}_n_ (**2**) and {[ZnL]∙0.5dmf∙1.5H_2_O}_n_ (**3**), as well as two heterometallic {[Zn_0.75_Cd_1.25_L_2_]∙dmf∙0.5H_2_O}_n_ (**4**) and {[MnZnL_2_]∙dmf∙3H_2_O}_n_ coordination polymers. Compound **1** represents a 1D chain, whereas **2**–**5** are isostructural and isomorphous two-dimensional structures. The entire series was characterized by IR spectroscopy, thermogravimetric analysis, single-crystal X-ray diffraction and emission measurements. 2D coordination polymers accommodate water and dmf molecules in their cage-shaped interlayer spaces, which are released when the samples are heated. Thus, three solvated crystals were degassed at two temperatures and their photoluminescent and adsorption–desorption properties were recorded in order to validate this assumption. Solvent-free samples reveal an increase in volume pore, adsorption specific surface area and photoluminescence with regard to synthesized crystals.

## 1. Introduction

Over the years, coordination polymers (CPs) have attracted interest, not only due to their interesting architectures and topologies, but also due to their diverse applications [1,2,3,4,5,6,7]. It is well known that CPs can be obtained by the combination of metal ions as a node and as the connecting rod can be employed by multidentate organic/inorganic ligands. The Schiff bases derived from 2,6-diacetylpyridine are good candidates for elaboration of magnetic homo- and/or heterometallic CPs [8,9,10,11,12]. The analysis of the Cambridge Structural Database (CSD, version 2020.2.0) revealed 20 transition metal CPs with this type of ligands [13]. In most of them, the metal atoms are interconnected by inorganic bridges, such as CN^−^ [9,12,14], N_3_^−^ [8], [Ni(CN)_4_]^2^^−^ [11,14,15], [Fe(CN)_6_]^3^^−^ [16,17,18] and [Mn(CN)_6_]^3^^−^ [19], but only in two examples the polymer dimensionality was realized by the ligand terminal arms, isonicotinoylhydrazone [10] and 2-aminobenzoylhydrazone [20]. The combination of various 2,6-diacetylpyridine Schiff bases and Zn(II)/Cd(II) metals led to 26 discrete coordination compounds with catalytic [21,22], magnetic [23], nonlinear optics [22], photoluminescent [24] and biological activity [25].

The 2,6-diacetylpyridine bis(nicotinoylhydrazone) ligand (H_2_L) can be a heptadentate ligand having both N- and O- donor set atoms, which are liable to coordinate to various transition metal atoms as neutral, anionic or cationic forms, presenting tetra-, penta- or hexadentate coordination fashions [26,27,28]. Our recent published tetranuclear coordination compound [Cu_4_(HL)_4_(OH)_2_](NO_3_)_2_∙6.75H_2_O [26] is the first example in which H_2_L shows unprecedented coordination mode involving one pyridine ring of nicotinamide moiety in bridging metal coordination.

The CSD analysis results and our own attempts [26,27,28] encouraged our interest to find the optimal synthetic method of obtaining Zn(II)/Cd(II) CPs based on H_2_L. Thus, three homo-, [CdL]_n_ (**1**), {[CdL]∙0.5 dmf∙H_2_O}_n_ (**2**) and {[ZnL]∙0.5 dmf∙1.5H_2_O}_n_ (**3**), and two heterometallic, {[Zn_0.75_Cd_1.25_L_2_]∙dmf∙0.5H_2_O}_n_ (**4**) and {[MnZnL_2_]∙dmf∙3H_2_O}_n_ (**5**), CPs have been prepared in similar solvothermal conditions, where H_2_L reveals its new coordination fashions. Alongside crystallographic studies, the Hirshfeld surface analysis was performed in order to examine intermolecular interactions within the crystal. Thermal and IR methods have been used to characterize crystals and to degas some CPs in an attempt to study their luminescent and adsorption–desorption properties. For this, the emission spectra of the Schiff base ligand and the synthesized compounds were also recorded and compared with degassed samples at different temperatures.

## 2. Results

### 2.1. Synthesis Aspects, Crystal Structure and Thermal Characterization

**Scheme 1:**
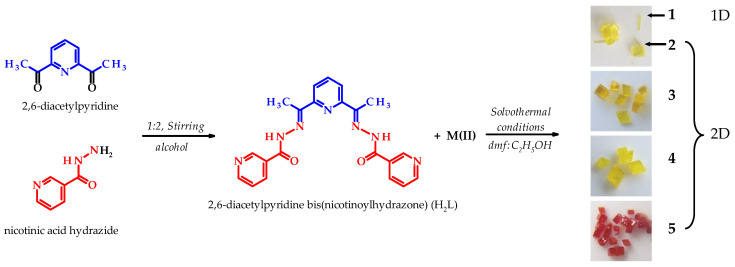
Schematic representation of the synthesis of ligand (H_2_L) and coordination compounds **1**–**5**.

The solvothermal synthesis between Zn(II)/Cd(II) nitrate and H_2_L ligand in the ethanol:dmf mixture at 120 °C led to elongated-prismatic, cubic and cuboid yellow crystals of CPs **1**–**3** (Scheme 1). The second transition metal, Cd(II) or Mn(II), has been added to the Zn(II)-H_2_L system, at the same solvent mixture and synthetic conditions to diversify the structures’ architectures and study their properties. Consequently, were obtained two new 2D heterometallic CPs {[Zn_0.75_Cd_1.25_L_2_]∙dmf∙0.5H_2_O}_n_ (**4**) and {[MnZnL_2_]∙dmf∙3H_2_O}_n_ (**5**). The compound **4** represents a yellow chromophore, while compound **5** is reddish colored. Regarding the presence of both transition metal ions in the new CPs, the qualitative report data for **4** and **5** have been registered (Figure S1).

It was noticed that all crystals of discussed compounds were obtained directly from solvothermal autoclaves in suitable shapes for X-ray analysis. The samples are stable at room temperature; however, they are characterized by total insolubility in both water and organic solvents. The current results and our recent study [26] revealed that the presence of the dmf solvent in solvothermal synthesis plays an essential role in the bideprotonation of the Schiff base ligand.

The IR spectra of the CPs confirm the organic ligand H_2_L coordination to metal ions. The characteristic absorption bands of the Schiff base are: ν(N-H) 3187 cm^−1^, ν(C=O)_(amid I)_ 1664 cm^−1^, δ(NH)+ν(CN)_(amid II)_ 1567 cm^−1^, δ(NH)+ν_as_(OCN)_(amid III)_ 1271 cm^−1^ and ν(C=N)_azomethine_ 1617 cm^−1^ [29,30]. In the IR spectra of the compounds **1**–**5**, the band ν(N-H) disappears, showing the bideprotonated coordination mode of the Schiff base ligand (Figure S2). Upon coordination, the absorption bands of amide I undergo significant shifts towards lower frequencies—in the range 1532–1520 cm^−1^. The ν(C-N) and ν_as_(OCN) bands, components of amide II and the corresponding amide III, are observed in the range 1583–1578 and 1266–1252 cm^−1^, respectively, while the ν(C=N)_azomethine_ band can be observed in the range 1597–1593 cm^−1^ [24,30,31,32,33,34]. Additionally, the shifting of the absorption band ν(C=C)+ν(C=N) of the pyridine ring from 1591 cm^−1^ in the ligand spectrum to 1583–1578 cm^−1^ in CPs is observed. These changes are caused by the coordination of the Schiff base through the carbonyl O atoms, azomethine and central heterocyclic N atoms [24,30,31,32,33,34], as well as by its dianionic character [31], which leads to the electron delocalization in the metallocycles. The bands of the M-N and M-O bonds could not be identified as in the case of analogue Zn(II) complexes [31], which shows that the frequencies of these bonds in such systems are manifested in the region 293–147 cm^−1^. The solvent guest molecules in **2**–**5** are represented by ν(C=O)_dmf_ band at 1671–1670 cm^−1^, and the range 3646–3337 cm^−1^ attributed to crystallization water molecules.

**Figure 1:**
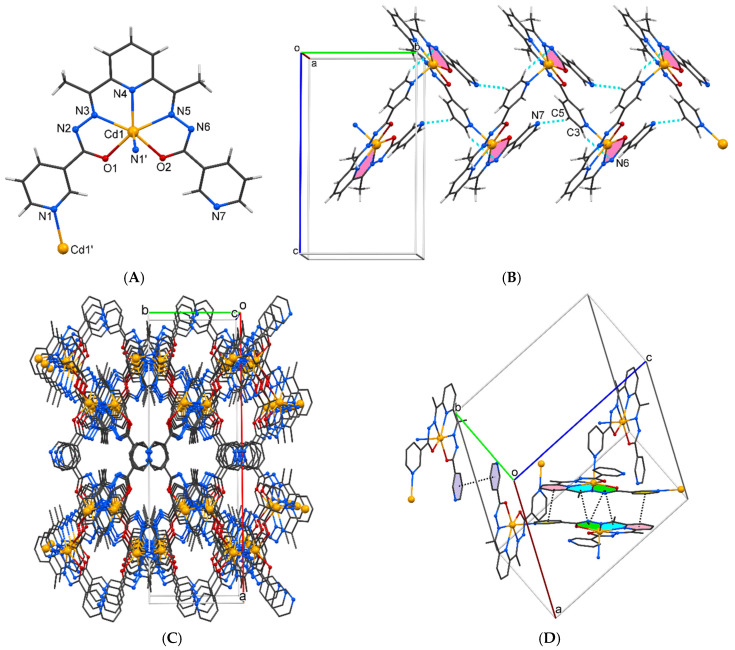
Coordination geometry of Cd(II) ion in **1** with partial atom labelling scheme (**A**). The 1D coordination polymer fragment in **1** with Cg(Cd1 > N5) colored in pink along *b* axe (**B**). Perspective view of crystal packing in **1** along *c* axe (**C**). The stacking of the chains linked by π∙∙∙π interactions between metalochelate-metalochelate and aromatic-aromatic systems. The Cg(Cd1 > N3) are shown green, Cg(Cd1 > N4)-cyan, Cg(N1 > C4)-yellow, Cg(N4 > C12)-rose and Cg(N7 > C17)-violet. H atoms are omitted for clarity (**D**). Element color scheme for figures (**A**–**C**): C—dark gray and H—light gray sticks; O—red, N—blue and Cd—orange balls.

Molecule **1**, [CdL]_n_, crystallizes in the monoclinic centrosymmetric *C*2/*c* (No 15) space group and presents a 1D coordination polymer. The asymmetric part of the unit cell contains one metal atom and one bideprotonated ligand (Figure 1A). Each Cd^2+^ is hexacoordinated (N_4_O_2_) with five positions occupied by one pyridine and two azomethine nitrogen atoms, and two carbonyl oxygen atoms from anionic ligand in the equatorial plane, while the sixth position is occupied by the nicotinic hydrazide nitrogen atom of the adjacent [CdL] entity, acting as a bridge. Thus, the geometry of cadmium cation can be described as a distorted pentagonal pyramid. Here, the 2,6-diacetylpyridine bis(nicotinoylhydrazone) is hexadentate (N_4_O_2_), and in addition to the five central donor atoms, only one terminal pyridine ring coordinates with the metal cation. The distortion from the ideal geometry is obviously shown by the angles between terminal pyridine N atom, cadmium, and the donor set of the L^2-^ anion (Table S1). This ligand shows an almost planar conformation proved by dihedral angles between the coordinated pyridine ring and both pyridine rings of nicotinamide moieties, coordinated and uncoordinated, equal to 7.4(2)° and 7.7(3)°. Four of the five angles subtended at Cd by atoms in the basal plane are slightly smaller than the ideal pentagonal pyramidal arrangement value, varying from 66.5(2)° to 68.5(1)°, while the fifth angle, O(1)-Cd(1)-O(2), is equal to 87.9(1)° (Table S1). The maximum deviation from the N_3_O_2_ least-squares calculated plane is 0.093(3) Å, with the Cd atom lying 0.208(3) Å above this plane. The CdL unit is joined by the nicotinic hydrazide arm of the Schiff base ligand of the adjacent unit in a zigzag-like coordination chain, with Cd∙∙∙Cd separation through the L linker equal to 8.001(8) Å and polymeric pitch of 8.900(9) Å. The coordination chain is reinforced via intrachain C-H∙∙∙N hydrogen bonds (Table S2) and C(4)-H(4)∙∙∙π stacking interactions between the pyridine terminal rings (Figure 1B) and the metalochelate centroid Cg(Cd1 > N5) of 3.118(7) Å. The resulted chains are further interlinked in a supramolecular network by π∙∙∙π stacking interactions and weak C-H∙∙∙N(O) hydrogen bonds (Figure 1C). Figure 1D represents all possible π∙∙∙π stacking interactions in the crystal between: (a) metalochelate-metalochelate systems, Cg(Cd1 > N3)∙∙∙Cg(Cd1 > N3)_1/2-*x*, 1/2-*y*, -*z*_ = 3.6125(2) Å and Cg(Cd1 > N3)∙∙∙Cg(Cd1 > N4)_1/2-*x*, 1/2-*y*, -*z*_ = 3.4348(2) Å; and (b) aromatic-aromatic systems, Cg(N1 > C4)∙∙∙Cg(N4 > C12)_1/2-*x*, 1/2-*y*, -*z*_ = 3.6414(2) Å and Cg(N7 > C17)∙∙∙Cg(N7 > C17)_-*x*, -*y*, -*z*_ = 3.5544(2) Å.

**Figure 2:**
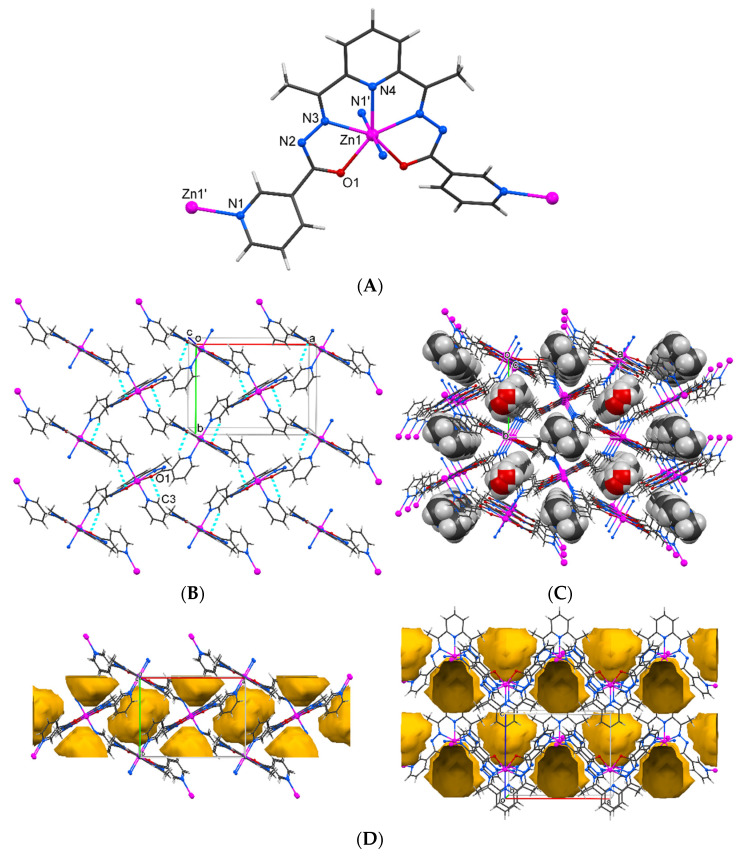
Coordination geometry of Zn(II) ion in **3** with partial atom labelling scheme (**A**). The 2D coordination polymer fragment viewed along the *c* axe in **3** (**B**). Fragment of crystal packing in **3** with the H_2_O and dmf inclusion in the interlayer spaces, solvent molecules being shown in the space-filling mode (**C**). The surface of solvent-accessible voids in crystal **3** generated by MERCURY after the solvent exclusion viewed along the *c* (left) and *b* (right) axes (**D**). Element color scheme: C—dark gray and H—light gray sticks; O—red, N—blue and Zn—magenta balls.

Compounds **2**–**5** are isostructural and isomorphous, crystallizing in the orthorhombic noncentrosymmetric *P*2_1_2_1_2 (No 18) space group with metal atoms and ligands residing on the twofold axis. Figure 2A displays the Zn(II) coordination polyhedron. In each compound, the metal atoms are seven-coordinated (N_5_O_2_) with a symmetrical pentagonal bipyramidal environment; in the equatorial plane, the L^2−^ dianion surrounds the metal atoms with one pyridine nitrogen (N4), two azomethine nitrogen atoms (N3 and N3′) and two carbonyl oxygen atoms (O1 and O1′), and the axial positions are occupied by two nitrogen atoms (N1 and N1′) from both pyridine rings of the nicotinamide moieties of adjacent ligands (Figure 2A). The L^2−^ shows a twisted conformation proved by the dihedral angles between coordinated and terminal pyridine rings, equal to 40.8(2)° in **2**, 40.0(2)° in **3**, 40.3(3)° in **4** and 40.0(4)° in **5**. All chelate bond angles around metal cations are equal to 66.5(1)° and 68.2(2)° in **2**, 68.5(1)° and 71.0(1)° in **3**, 66.5(2)° and 68.9(2)° in **4**, and 68.1(1)° and 70.5(2)° in **5**, while the nonchelate angles O(1)-M(1)-O(1′) are 91.2(2)° in **2**, 81.0(2)° in **3**, 89.7(3)° in **4** and 83.0(2)° in **5**. The sum of all these angles is 360.2° for Zn and Mn/Zn, 360.5° for Zn/Cd and 360.2° for Cd, proving the perfect planarity of all structures. The apical nitrogen and metal atoms form 177.0(3), 178.5(2), 176.8(4) and 177.8(3)° angles in **2**–**5**, showing a slight deviated linear arrangement (Table S1). The pyridines of hydrazide moieties associate with two adjacent metal atoms in a 2D wave-like layer (Figure 2B), with the diagonal dimensions of the rhombohedral meshes of 9.4836(4) × 13.6165(4) Å, 9.4383(3) × 12.4717(3) Å, 9.4973(4) × 13.3860(6) Å and 9.4825(6) × 12.6536(8) Å for **2**–**5**, respectively. The M∙∙∙M separations across the L ligand are 8.7527(4), 8.4625(5), 8.6934(5) and 8.5122(8) Å in **2**–**5**, respectively. The coordination layers are reinforced via an intralayer C(3)-H∙∙∙O(1) hydrogen bond (Table S2). All four solids accommodate polar protic and aprotic solvent molecules (H_2_O and dmf) in their compartments, bonded through C-H∙∙∙O hydrogen bonds with pyridine rings of nicotinamide moieties of L^2−^ ligand (Figure 2C, Table S2).

The structures of **4** and **5**, though they are isostructural and isomorphous to those of **2** and **3**, have some minor differences. If in homometallic CPs the solvent molecules are bonded with the polymeric layers by C-H∙∙∙O H-bonds (Table S2), then in compounds **4** and **5,** in addition to the intermolecular contact, the dmf molecule is also joined by C-H∙∙∙π interactions with the nicotinamide aromatic system (C-H∙∙∙Cg(N1 > C4) is equal to 3.99(3) Å in both structures). The volume, occupied by solvent molecules, according to PLATON calculations for simulated solvent-free networks, is 334.4 Å^3^ (~26.5% of the total unit cell volume) in **2**, 299.9 Å^3^ (~25.1%) in **3**, 330.4 Å^3^ (~26.4%) in **4** and 302.8 Å^3^ (~25%) in **5**. These values indicate that all 2D crystals have high solvent uptakes (Figure 2D).

**Figure 3:**
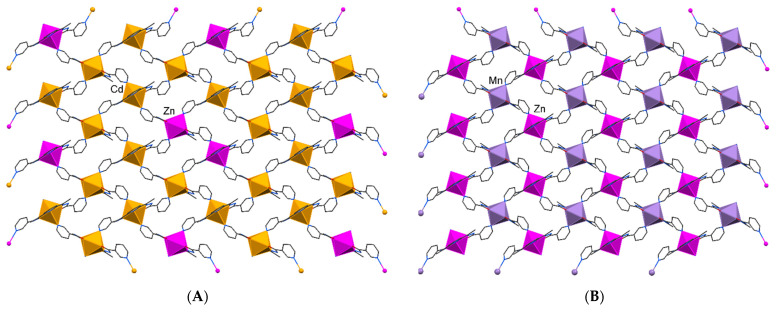
View of sequence of metals in CPs **4** (**A**) and **5** (**B**), respectively, found by Monte Carlo simulation. Metal atoms are shown in polyhedral fashion.

It was found that in the crystal structures of coordination polymers **4** and **5** the ratio in unit cell of Cd:Zn and Mn:Zn were 1.25:0.75 and 1:1, respectively. Therefore, the Monte Carlo generator of Special Quasirandom Structures (mcsqs) code [35] was used to generate the Special Quasirandom Structures (SQS) to find the sequence of metals in the CPs **4** and **5** within the aforecited ratio (Figure 3). This method is the best periodic supercell approximation to find the true disordered state for a given number of atoms per supercell. This code is implemented in Alloy Theoretic Automatic Toolkit and it based on a Monte Carlo simulated annealing loop with an objective function that tends to perfectly match the maximum number of correlation functions. The chosen method optimizes the shape of the supercell jointly with the occupation of the atomic sites and thus ensuring the configurational space searched is exhaustive and not biased by a pre-specified supercell shape. To generate SQS’s, 2 × 2 × 2 (768 atoms) and 1 × 1 × 1 (96 atoms) supercells were used for compounds **4** and **5**, respectively.

**Figure 4:**
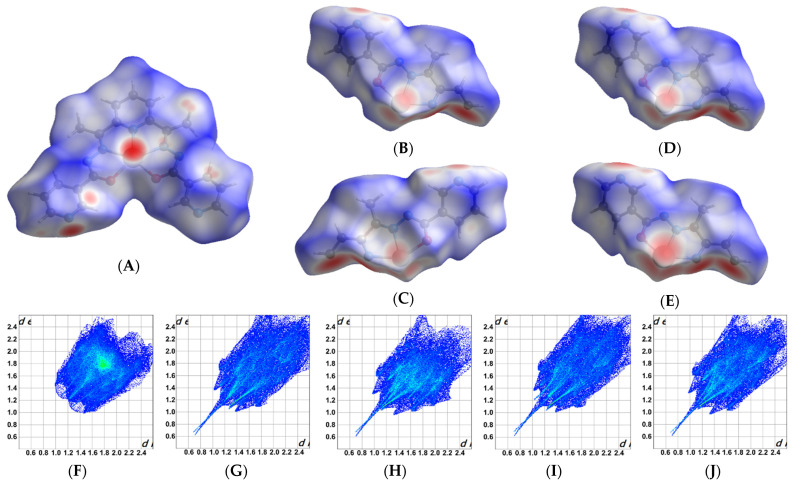
Hirshfeld surfaces of the asymmetric units in **1**–**5** (**A**–**E**) mapped over d_norm_ and their 2D fingerprint plots (**F**–**J**), respectively.

In order to elucidate the weak intermolecular interactions, the 3D Hirshfeld surfaces (HS) analysis of molecular units in **1**–**5** was chosen as the most convenient method, which can be represented by normalized surfaces, as well as by 2D fingerprint plots of any possible types of short contacts. HS of asymmetric units in **1**–**5** have been mapped with normalized contact distance d_norm_ (Table S4) and illustrated in Figure 4, in which the red spots relate to the dominant intermolecular interactions in the crystals. The full 2D fingerprint plots for asymmetric unit in coordination polymers **1**–**5** are consistent with HS and are represented as plots of d_i_ versus d_e_ (the distances from the HS to the the nearest atom inside and outside the surface) in which is clearly observed the different distribution of various interactions in the crystal structures (Figure 4F–J). The quantification of these intermolecular interactions was presented as a chart (Figure S3) and according to it in all molecules the H∙∙∙H short intermolecular contacts have the highest contribution to the total HS. It can be seen in the middle of the scattered points in the fingerprint maps, ranging from 26.6% in **2** and **4** to 38.5% in **1**. The next contacts with large surface area in 2D fingerprint plots are C∙∙∙H/H∙∙∙C, which can be observed as two partially wide wing-like peaks and usually are attributed to C-H∙∙∙π interactions. Other significant weak interactions observed in all crystals are N∙∙∙H/H∙∙∙N and O∙∙∙H/H∙∙∙O, the sums of which fall in the range of 23–24.5% of total surface area in 2D fingerprint plots. The C∙∙∙C contacts, which are an estimation of the π∙∙∙π stacking interactions, cover around 5.8% in **1**, 3.1% in **3**, and ~8.4% in **2**, **4** and **5**. Despite that these contacts in **2**, **4** and **5** cover a larger surface of the 2D fingerprint plots, the π∙∙∙π interactions in **1** are stronger (ranging 3.4348(2)-3.6414(2) Å), in comparison to the compounds **2**, **4** and **5** (the minimum values are 5.095(5), 5.065(6) and 4.980(5) Å). The shape index surface of **1** clearly shows the signature of C∙∙∙C interactions that are recognized by the red and blue triangles (Figure S4). The HS of the metal atoms in all compounds show deep-red regions for metal atoms, highlighting the short Me∙∙∙N contacts, ranging from 3.8 % in **1** to 7.4 % in **2**.

In addition to visualizing, exploring, and quantifying intermolecular interactions in the crystal lattice of all compounds, we obtained quantitative measures of HS for molecules **1**–**5** (Table S4). The lower Hirshfeld volumes and surface areas in **2**–**5** indicate that these molecules have a more crowded environment in comparison with **1**, which is evident in its 2D fingerprint plot by the compact pattern of this molecule. Globularity values of all coordination polymers show that all of them deviate from a spherical surface. The anisotropy of the surface is expressed by the asphericity, which can take a value of zero for an isotropic surface, 0.25 for an oblate, and 1.0 for an elongated object. The asphericity values of present CPs show their deviation from symmetry.

Thermal analysis of compounds **2**–**4** showed that the first decomposition step begins at 260 °C (Figure S5), representing a thermal degradation by hydrazine bond (-N-N=) splitting [36], which leads to the elimination of solvent guest molecules. After the first step of decomposition, the intermediate products stay stable up to 380 °C, indicating a strong connection between the metal and the oxygen and nitrogen atoms. Further increasing of the temperature leads to the decomposition of the organic residue, a process which ends at 560 °C and, as a result, the corresponding metal oxides are formed.

### 2.2. Photoluminescent Properties of CPs ***1**–**5***

**Figure 5:**
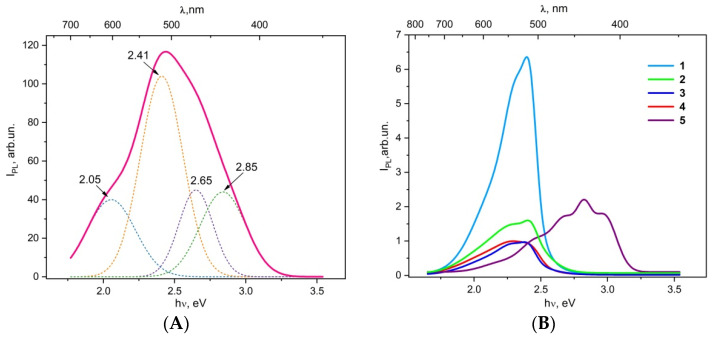
Solid-state PL emission spectra (λ_ex_ = 337 nm) of H_2_L ligand (**A**) and CPs **1**–**5** (**B**). The deconvolution of Gaussian resolution functions are shown by thin dash lines with the presentation of the maximum peaks in eV.

The photoluminescence (PL) properties of all studied CPs and H_2_L ligand (Figure 5) were studied in the solid-state at room temperature, λ_ex_ = 337 nm, in the wavelength region 350–750 nm. The shapes of the emission curves for all samples indicate the superpositions of several radiative processes. The Gaussian function was used to deconvolute the spectrum into separate bands. As a result of the ligand PL spectra simulation, we obtained the superpositions of four Gaussian curves with maxima at 2.05 (604.3 nm), 2.41 (514 nm), 2.65 (467.5 nm), and 2.85 eV (434.7 nm). The PL intensity of the ligand is more than an order of magnitude higher than that observed in the studied complexes.

Along with the peak 2.41 eV, the PL spectra of the complexes exhibit peaks at 2.3 eV (538.7 nm), which are absent in the ligand spectrum. In **1**, **2** and **3**, the peaks at 2.3 and 2.4 eV are of the same magnitude order, while in sample **4** the peak at 2.3 eV slightly dominates. Compound **1** is solvent-free and its crystal packing presents strong π∙∙∙π stacking interactions between metalochelate and aromatic systems, which most likely enhance the PL properties of the 1D CP with respect to isostructural and isomorphous layered compounds. From layered **2**–**4** CPs, both homo- and heterometallic, the Cd(II) compound **2** has the most intense PL property. Meanwhile, the heterometallic CP **4** shows an intensity much closer to the Zn(II) CP, which suggests that PL is quenched, explained by the presence of two metal ions (Cd(II), Zn(II)), similar to the phenomenon of antagonism, when one substance (in our case, metal ion) suppresses or negates the effect on the other. Taking into account that Zn(II) and Cd(II) ions do not present emissive electronic transitions, compounds **2**–**4** exhibit green fluorescence which are assigned to ligand-based luminescence.

In CP **5** the PL appears in the short-wavelength region of the spectrum and its complex shape represents a superposition of six peaks. The most intense peaks are at 2.7 (458.8 nm), 2.8 (442 nm), and 3.0 eV (413 nm). The emission spectrum of this heterometallic CP based on Zn(II) and Mn(II) is blue-shifted in comparison to its isostructural and isomorphous compounds, indicating that blue-violet PL of **5** originates from the metal-centred electronic transitions influenced by the coordination environment of Mn^2+^ ions [37,38].

### 2.3. Investigation of Crystal Properties after Guest Molecules Degassing

**Figure 6:**
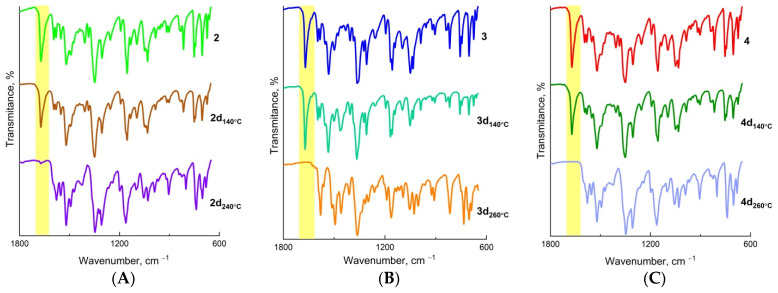
Comparable IR spectra for CPs **2**–**4** (**A**–**C**), and desolvated samples with the coloration of the ν(C=O) band.

Thermal analysis of the CPs **2**–**4** indicated that the solvent molecules were removed at a surprisingly high temperature (240–260 °C) when the ligand thermal degradation began. These observations indicate that water and dmf molecules were accommodated in their compartments and when heated these spaces are freed. Thus, to prove this assumption, nitrogen adsorption isotherms were measured at 77 K on degassed samples at two temperatures: 140 °C to remove molecules adsorbed from the air (getting **2d_140°C_**, **3d_140°C_** and **4d_140°C_** samples) and 240/260 °C to eliminate the solvent guest molecules (samples **2d_260°C_**, **3d_240°C_** and **4d_260°C_**). The crystals of all CPs were first heated to 240 °C and only crystals of **2** lost guest molecules, while samples **3** and **4** were further heated to 260 °C. Removal of dmf molecules was determined by IR spectroscopy, evidencing the absence of the ν(C=O) band at ~1670 cm^−1^ (Figure 6). All degassing crystals kept their shapes and crystallinity after heating at 140 °C, becoming brighter, but lost their lustre after degassing at higher temperatures (Figure S6).

**Figure 7:**
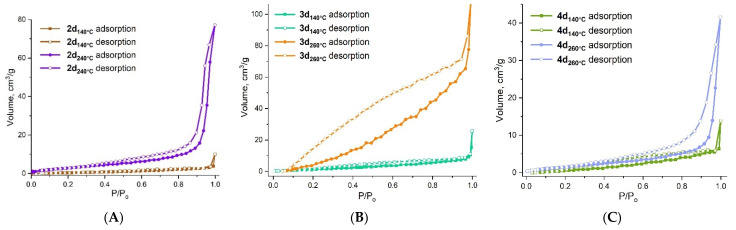
Adsorption–desorption isotherms of N_2_ at 77 K for: **2d_140°C_** and **2d_260°C_** (**A**); **3d_140°C_** and **3d_240°C_** (**B**); **4d_140°C_** and **4d_260°C_** (**C**).

The obtained results clearly show that there is a significant increase in volume pores and specific surface area (Figure 7, Table S4). At the same time, the appearance of hysteresis rings for degassed samples at 240/260 °C indicates the presence of mesopores. The pore volume distribution curves according to the radius (Figure S7) also indicate the existence of pores in the degassed samples at 240/260 °C. For crystals of **3**, the pores have a radius of 3 nm, while those of **2** and **4** were found to have radii of 12 nm.

**Figure 8:**
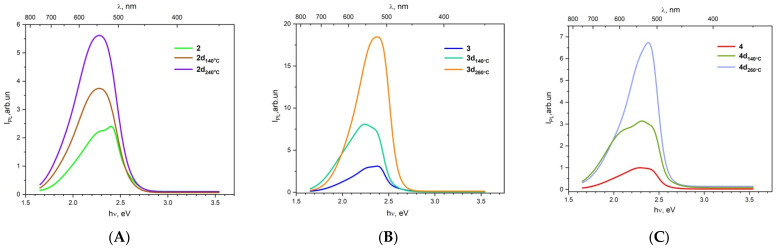
Comparable solid-state emission plots (λ_ex_ = 337 nm) for compounds **2**–**4** (**A**–**C**), and degassed samples.

The PL intensity of the degassing samples increases by several times with regard to synthesized crystals and in consequence, the PL of the long-wavelength wing (2.3 eV) becomes much more pronounced (Figure 8). Intensification of this peak leads to shape changes in the PL spectrum and the most obvious is especially for CPs **2** and **3**. These results show that obtained materials have sensitivity to the removal of guest molecules and can be recommended as sensors, thus extending the Zn(II)/Cd(II) family [39,40,41,42] of CPs with impressive sorption-luminescent properties.

## 3. Materials and Methods

The ligand H_2_L was prepared by the condensation of the 2,6-diacetylpyridine and nicotinic acid hydrazide according to synthetic methods described earlier [43]. Both reagents and solvents were of analytical grade and were purchased from Fluka Chemie AG (Buchs SG, Switzerland) and Sigma-Aldrich (St. Louis, MO, USA).

Elemental analysis was performed on an Elementar Analysen systeme GmbH Vario El III elemental analyser (Elementar Analysensysteme GmbH, Hanau, Germany). The IR spectra were obtained in Vaseline oil and ATR on a FT IR Spectrum-100 Perkin Elmer spectrometer in the range of 400–4000 cm^−1^ (PerkinElmer Life & Analytical Sciences, Beaconsfield, UK). Samples **4** and **5** were analysed with the elemental analyzer spectrometer Energy Dispersive X-Ray Fluorescence (X-Calibur-Xenemetrix, Migdal Haemek, Israel) looking for Zn, Cd and/or Mn content. Thermal analysis was performed on Derivatograph Q-1000 system in nitrogen atmosphere at a heating rate of 10 °C/min in the temperature range of 25–1000 °C and thermogravimetric (TG), derivative weight loss (DTG) and differential thermal (DTA) curves were simultaneously registered.

Structure and adsorption parameters of samples were obtained from nitrogen adsorption isotherms at 77 K. The adsorption–desorption isotherms were measured using Autosorb-1-MP (Quantachrome, Boynton Beach, FL, USA), with prior degassing at different temperatures for 12 h. The specific surface area was calculated using the Brunauer-Emmett-Teller (BET) equation. The total pore volume was calculated by converting the amount of N_2_ gas adsorbed at a relative pressure of 0.99 to equivalent liquid volume of the adsorbate (N_2_).

Photoluminescent emission spectra of studied single crystals excited with nitrogen laser λ_ex_ = 337 nm, duration = 15 ns, time repetition 50 Hz were collected at room temperature.

### 3.1. Synthesis of Coordination Polymers

The mixture of H_2_L (0.038 g, 0.1 mmol), metal salt (Zn(NO_3_)_2_·6H_2_O, 0.030 g; Cd(NO_3_)_2_·4H_2_O, 0.031 g, 0.1 mmol; Zn(NO_3_)_2_·6H_2_O, 0.015 g, Cd(NO_3_)_2_·4H_2_O, 0.016 g; ZnSO_4_·7H_2_O, 0.014 g, MnSO_4_·5H_2_O, 0.012 g, 0,05 mmol), dimethylformamide (5 mL) and ethanol (3 mL) was sealed in a Teflon-lined autoclave and heated at 120 °C for 48 h under autogenous pressure. H_2_O (1 mL) was added to sulphate anions-containing autoclaves, i.e., in reaction of compound **5**. The cooling was realized at the rate of 0.06 °C/min to room temperature. After three days, the zinc (**3**), zinc/cadmium (**4**) and zinc/manganese (**5**) complexes were obtained as yellow (**3** and **4**) and red crystalline solids (**5**). In the case of cadmium, both yellow elongated-prismatic (**1**) and yellow cuboid (**2**) crystals were observed in the solution. All compounds were collected by filtration, washed with ethanol at room temperature and then dried in air. Crystals of **1** and **2** were separated manually and used for further analysis.

Data for [CdL]_n_ (**1**): Anal. Calc. for C_21_H_17_CdN_7_O_2_, %: C, 49.28; H, 3.35; N, 19.16. Found, %: C, 49.35; H, 3.40; N, 19.20. IR (cm^−1^): 1520 ν(C=O)_(amide I, complex)_; 1578 ν(CN)_(of amide II)_; 1266 ν_as_(OCN)_(of amide III)_; 1597 ν(C=N)_azomethine_.

Data for {[CdL]·0.5dmf·H_2_O}_n_ (**2**): Anal. Calc. for C_45_H_45_Cd_2_N_11_O_11_, %: C, 47.38; H, 3.98; N, 13.51. Found, %: C, 47.35; H, 4.01; N, 13.44. IR (cm^−1^): 3600 ν(OH)(H_2_O); 1520 ν(C=O)_(amide I, complex)_; 1671 ν(C=O)_(amide I, dmf)_; 1580 ν(CN)_(of amide II)_; 1256 ν_as_(OCN)_(of amide III)_; 1594 ν(C=N)_azomethine_.

Data for {[ZnL]·0.5dmf·1.5H_2_O}_n_ (**3**): Yield (based on ligand): 66%. Anal Calc. for C_45_H_51_Zn_2_N_15_O_8_, %: C, 50.95; H, 4.85; N, 19.81. Found, %: C, 50.90; H, 4.81; N, 19.79. IR (cm^−1^): 3676 ν(OH)(H_2_O); 1532 ν(C=O)_(amide I, complex)_; 1670 ν(C=O)_(amide I, dmf)_; 1583 ν(CN)_(of amide II)_; 1252 ν_as_(OCN)_(of amide III)_; 1596 ν(C=N)_azomethine_.

Data for {[Zn_0.75_Cd_1.25_L_2_]·dmf∙0.5H_2_O}_n_ (**4**): Yield (based on ligand): 85%. Anal. Calc. for C_45_H_42_Cd_1.25_Zn_0.75_N_15_O_5.50_, %: C, 50.49; H, 3.95; N, 19.63. Found, %: C, 50.60; H, 4.05; N, 19.70. IR (cm^−1^): 3416 ν(OH)(H_2_O); 1520 ν(C=O)_(amide I, complex)_; 1671 ν(C=O)_(amide I, dmf)_; 1580 ν(CN)_(of amide II)_; 1254 ν_as_(OCN)_(of amide III)_; 1593 ν(C=N)_azomethine_.

Data for {[MnZnL_2_]·dmf∙3H_2_O}_n_ (**5**): Yield (based on ligand): 29%. Anal. Calc. for C_45_H_47_MnZnN_15_O_8_, %: C, 51.66; H, 4.53; N, 20.08. Found, %: C, 51.50; H, 4.60; N, 20.15. IR (cm^−1^): 3337 ν(OH)(H_2_O); 1525 ν(C=O)_(amide I, complex)_; 1670 ν(C=O)_(amide I, dmf)_; 1583 ν(CN)_(of amide II)_; 1258 ν_as_(OCN) _(of amide III)_; 1595 ν(C=N)_azomethine_.

### 3.2. Crystallographic Data Collection and Refinements

Single crystal X-ray diffraction measurements for **1**–**5** were performed at room temperature on a Xcalibur E CCD diffractometer (Abingdon, Oxfordshire, United Kingdom) equipped with a CCD area detector and a graphite monochromator utilizing MoKα radiation at room temperature. All the calculations to solve the structures and to refine the models proposed were carried out with the programs SHELXS97 and SHELXL2014 [44,45]. Hydrogen atoms bonded to C atoms were refined using a riding-model approximation, with C-H = 0.93 and 0.96 Å for CH (aromatic) and CH_3_ groups, respectively, which were fixed with U_iso_ (H) = 1.2U_eq_ (C) and U_iso_ (H) = 1.5U_eq_ (CH_3_). The dmf guest molecules were refined with an occupation factor of 0.25 in **2**–**5**. For the dmf molecules in compound **4** and **5**, no hydrogen atoms were found. In CPs **3**–**5**, the lattice water molecules were refined with a partial occupancy factor: in **2**, 0.15 and 0.35; in **3**, 0.25 for all three molecules; in **4**, 0.125 for one water molecule; in **5**, the molecules were refined with 0.25 and 0.5 partial occupancy factors. The O3W water molecule in **3** does not participate in hydrogen bonds with one proton. The crystal data and structure refinement details for compounds **1**–**5** are presented in Table 1, while Tables S1 and S2 give selected geometric parameters. The figures were produced using the MERCURY program [46]. The solvent-accessible voids were calculated using PLATON [47]. All geometrical data in text were calculated using SHELXL2014, MERCURY and/or PLATON programs. Crystallographic data of **1**–**5** were deposited with the Cambridge Crystallographic Data Centre and allocated the deposition numbers CCDC 2070426-2070430.

### 3.3. Computational Details

The Hirshfeld surface analysis and 2D fingerprint plots for all compounds were performed using *CrystalExplorer* 17.5 (Version 17.5) [48] program, specifically to examine and visualize different intermolecular contacts within the crystals, as well as quantitative measures of Hirshfeld surfaces for CPs molecules.

The method of Special Quasirandom Structures (SQS) [35] provides the search of true disordered state for a given number of atoms per supercell. Two codes from the Alloy Theoretic Automated Toolkit (ATAT, Version 3.04) package were used to generate SQS: corrdump and mcsq. The code corrdump calculates symmetries and clusters. The cut-off value of 3.8 Å was chosen to define the shells of nearest neighbors to calculate the correlation functions. The mcsqs code, based on a Monte Carlo algorithm, was used to find a perfectly matched SQS. Two files provided the input for this code: (i) the file containing the random state; (ii) the cluster file generated by corrdump code.

## 4. Conclusions

The solvothermal synthesis was chosen as the most optimal method of Zn(II) and Cd(II) CPs preparation based on the Schiff base H_2_L and led to the obtention of three homo- and two heterometallic materials. One of them represents a 1D coordination chain, while the rest are 2D isostructural and isomorphous coordination layers. In addition to the dimensionality, compounds are also distinguished by metal coordination polyhedrons (N_4_O_2_ in 1D and N_5_O_2_ in 2D) and the Schiff base coordination mode to M(II) atoms. The 2D CPs accommodate solvent guest molecules in the interlayer spaces and the solvent-accessible voids adopt a discrete cage shape. The green ligand-based emission for all Zn(II) and Cd(II) CPs and blue-violet photoluminescence for Zn(II) Mn(II) heterometallic compound were registered. The association of thermal and IR methods was used to degas the accommodated guest samples at different temperatures as well as to detect the removal of molecules. The degassing crystals revealed a significant increase in volume pores and specific surface area, as well as PL emission with respect to synthesized ones. These findings make the layered compounds excellent small solvent sensor materials, expanding the family of CPs with interesting properties.

## Figures and Tables

**Table 1:** Crystal and structure refinement data for **1**–**5**.

Compound	1	2	3	4	5
Formula	C_21_H_17_Cd_1_N_7_O_2_	C_45_H_45_Cd_2_N_11_O_11_	C_45_H_51_Zn_2_N_15_O_8_	C_45_H_42_Cd_1.25_Zn_0.75_N_15_O_5.5_	C_45_H_47_MnZnN_15_O_8_
Formula weight	511.82	566.38	530.37	1070.46	1046.28
Crystal system	Monoclinic	Orthorhombic	Orthorhombic	Orthorhombic	Orthorhombic
Space group	*C*2/*c*	*P*2_1_2_1_2	*P*2_1_2_1_2	*P*2_1_2_1_2	*P*2_1_2_1_2
*a* (Å)	28.6789(18)	13.6165(4)	12.4717(3)	9.4973(4)	9.4825(6)
*b* (Å)	8.9005(6)	9.4836(4)	9.4383(3)	13.3860(6)	12.6536(8)
*c* (Å)	16.0789(11)	9.7867(3)	10.1540(3)	9.8617(4)	10.0787(8)
β (°)	104.375(7)	90	90	90	90
*V* (Å^3^)	3975.8(5)	1263.79(8)	1195.24(6)	1253.71(9)	1209.32(14)
*Z*	8	2	2	1	1
ρ_calc_ (g cm^−3^)	1.710	1.488	1.474	1.418	1. 437
μ_Mo_ (mm^−1^)	1.134	0.904	1.073	0.948	0.825
*F*(000)	2048	572	550	544	541
Crystal size (mm)	0.42 × 0.14 × 0.08	0.40 × 0.30 × 0.20	0.30 × 0.25 × 0.25	0.22 × 0.22 × 0.22	0.38 × 0.38 × 0.22
Reflections collected/unique	6602/3698 (*R*_int_ = 0.0587)	3172/2241 (R_int_ = 0.0225)	2963/1841 (*R*_int_ = 0.0237)	3151/2040 (*R*_int_ = 0.0303)	3062/1812 (*R*_int_ = 0.0332)
Completeness (%)	99.8	99.6	99.2	98.9	99.2
Reflections with *I* > 2σ(*I*)	2471	2059	1686	1745	1596
Parameters	282	161	166	161	161
GOF	1.000	1.007	1.000	1.007	1.008
*R_1_*, *wR_2_* (*I* > 2σ(*I*))	0.0532, 0.0997	0.0367, 0.0995	0.0367, 0.0960	0.0453, 0.1175	0.0472, 0.1215
*R_1_*, *wR_2_* (*all data)*	0.0876, 0.1156	0.0414, 0.1038	0.0417, 0.0994	0.0562, 0.1268	0.0563, 0.1287
Δρ_max_/Δρ_min_ (*e*⋅Å^−3^)	0.771/−0.807	0.976/−0.463	0.476/−0.454	0.775/−0.305	0.601/−0.314

## Data Availability

Not applicable.

## Supplementary Materials

The following are available online, Figure S1: Qualitative report data for compounds **4** and **5**, Figure S2: The contribution of Amide vibrational modes in the IR spectra of H_2_L and CPs **1**–**5** in the interval 600–1800 cm^−1^ and their representation on the ligand structure, Table S1: Selected bond lengths (Å) and angles (°) in coordination metal environment in **1**–**5**, Table S2: Hydrogen bond distances (Å) and angles (°) for compounds **1**–**5**, Figure S3: Relative contributions of various intermolecular contacts to the Hirshfeld surface area in compounds **1**–**5**, Figure S4: Hirshfeld surface under the shape index function (from −1.0 (red) to 0.995 (blue) Å), demonstrating the presence of stacking interactions in the crystal of compound **1**, Table S3: Hirshfeld surface properties for compounds **1**–**5**, Figure S5: Thermoanalytical curves of compounds **2**–**4** (A-C), as well as comparative TG (D), Figure S6: Photos of synthesized **2**–**4** and degassed crystals at various temperatures, demonstrating shape stability and loss of brightness upon removal of guest solvent molecules, Table S4: Adsorption parameters of studied samples, Figure S7: Pore size distribution of degassing samples at different temperatures.

## Sample Availability

Samples of the compounds **1**–**5** are available from the authors.

## References

[1] Batten S.R., Neville S.M., Turner D.R. (2009). Coordination Polymers: Design. Analysis and Application.

[2] Ortiz O.L., Ramirez L.D. (2012). Coordination Polymers and Metal Organic Frameworks.

[3] García H., Navalón S. (2018). Metal-Organic Frameworks: Applications in Separations and Catalysis.

[4] MacGillivray L.R., Lukehart C.M., MacGillivray L.R., Lukehart C.M. (2014). Metal-Organic Framework Materials.

[5] Janiak C. (2003). Engineering coordination polymers towards applications. Dalton Trans..

[6] Heine J., Muller-Buschbaum K. (2013). Engineering metal-based luminescence in coordination polymers and metal–organic frameworks. Chem. Soc. Rev..

[7] Zhang X., Wang W., Hu Z., Wang G., Uvdal K. (2015). Coordination polymers for energy transfer: Preparations, properties, sensing applications, and perspectives. Coord. Chem. Rev..

[8] Bazhenova T.A., Mironov V.S., Yakushev I.A., Svetogorov R.D., Maximova O.V., Manakin Y.V., Kornev A.B., Vasiliev A.N., Yagubskii E.B. (2020). End-to-end azido-bridged lanthanide chain complexes (Dy, Er, Gd, and Y) with a pentadentate Schiff-base [N_3_O_2_] ligand: Synthesis, structure, and magnetism. Inorg. Chem..

[9] Pichon C., Elrez B., Béreau V., Duhayon C., Sutter J.-P. (2018). From heptacoordinated Cr^III^ complexes with cyanide or isothiocyanate apical groups to 1D heterometallic assemblages with all-pentagonal-bipyramid coordination geometries. Eur. J. Inorg. Chem..

[10] Naskar S., Corbella M., Blake A.J., Chattopadhyay S.K. (2007). Versatility of 2,6-diacetylpyridine (dap) hydrazones in generating varied molecular architectures: Synthesis and structural characterization of a binuclear double helical Zn(II) complex and a Mn(II) coordination polymer. Dalton Trans..

[11] Bar A.K., Gogoi N., Pichon C., Durga Prasad Goli V.M.L., Thlijeni M., Duhayon C., Suaud N., Guihery N., Barra A.-L., Ramasesha S. (2017). Pentagonal bipyramid Fe^II^ complexes: Robust ising-spin units towards heteropolynuclear nanomagnets. Chem. Eur. J..

[12] Bretosh K., Béreau V., Duhayon C., Pichon C., Sutter J.-P. (2020). A ferromagnetic Ni(II)–Cr(III) single-chain magnet based on pentagonal bipyramidal building units. Inorg. Chem. Front..

[13] Groom C.R., Bruno I.J., Lightfoot M.P., Ward S.C. (2016). The Cambridge structural database. Acta Crystallogr. B.

[14] Pichon C., Suaud N., Duhayon C., Guihéry N., Sutter J.-P. (2018). Cyano-bridged Fe(II)–Cr(III) single-chain magnet based on pentagonal bipyramid units: On the added value of aligned axial anisotropy. J. Am. Chem. Soc..

[15] Bar A.K., Pichon C., Gogoi N., Duhayon C., Ramasesha S., Sutter J.-P. (2015). Single-ion magnet behaviour of heptacoordinated Fe(II) complexes: On the importance of supramolecular organization. Chem. Commun..

[16] Dey M., Sarma B., Gogoi N. (2014). Coligand promoted controlled assembly of hierarchical heterobimetallic nitroprusside based aggregates. Z. Anorg. Allg. Chem..

[17] Kopotkov V.A., Sasnovskaya V.D., Korchagin D.V., Morgunov R.B., Aldoshin S.M., Simonov S.V., Zorina L.V., Schaniel D., Woike T., Yagubskii E.B. (2015). The first photochromic bimetallic assemblies based on Mn(III) and Mn(II) Schiff-base (salpn, dapsc) complexes and pentacyanonitrosylferrate. CrystEngComm.

[18] Zorina L.V., Simonov S.V., Sasnovskaya V.D., Talantsev A.D., Morgunov R.B., Mironov V.S., Yagubskii E.B. (2019). Slow magnetic relaxation, antiferromagnetic ordering, and metamagnetism in Mn^II^(H_2_dapsc)-Fe^III^(CN)_6_ chain complex with highly anisotropic Fe-CN-Mn spin coupling. Chem. Eur. J..

[19] Sasnovskaya V.D., Kopotkov V.A., Talantsev A.D., Morgunov R.B., Yagubskii E.B., Simonov S.V., Zorina L.V., Mironov V.S. (2017). Synthesis, structure, and magnetic properties of 1D {[Mn^III^(CN)_6_][Mn^II^(dapsc)]}_n_ coordination polymers: Origin of unconventional single-chain magnet behaviour. Inorg. Chem..

[20] Naskar S., Mishra D., Chattopadhyay S.K., Corbella M., Blake A.J. (2005). Versatility of 2,6-diacetylpyridine (dap) hydrazones in stabilizing uncommon coordination geometries of Mn(II): Synthesis, spectroscopic, magnetic and structural characterization. Dalton Trans..

[21] Li Z.-W., Wang X., Wei L.-Q., Ivanović-Burmazović I., Liu G.-F. (2020). Subcomponent self-assembly of covalent metallacycles templated by catalytically active seven-coordinate transition metal centers. J. Am. Chem. Soc..

[22] Hu G., Miao H., Mei H., Zhou S., Xu Y. (2016). Two novel bi-functional hybrid materials constructed from POMs and a Schiff base with excellent third-order NLO and catalytic properties. Dalton Trans..

[23] Shen F.-X., Li H.-Q., Miao H., Shao D., Wei X.-Q., Shi L., Zhang Y.-Q., Wang X.-Y. (2018). Heterometallic M^II^Ln^III^ (M=Co/Zn; Ln=Dy/Y) complexes with pentagonal bipyramidal 3d centers: Syntheses, structures, and magnetic properties. Inorg. Chem..

[24] Konar S., Jana A., Das K., Ray S., Chatterjee S., Golen J.A., Rheingold A.L., Kar S.K. (2011). Synthesis, crystal structure, spectroscopic and photoluminescence studies of manganese(II), cobalt(II), cadmium(II), zinc(II) and copper(II) complexes with a pyrazole derived Schiff base ligand. Polyhedron.

[25] Rodriguez-Arguelles M.C., Ferrari M.B., Fava G.G., Pelizzi C., Tarasconi P., Albertini R., Dall’Aglio P.P., Lunghi P., Pinelli S. (1995). 2,6-Diacetylpyridine bis(thiosemicarbazones) zinc complexes: Synthesis, structure, and biological activity. J. Inorg. Biochem..

[26] Danilescu O., Bulhac I., Shova S., Novitskii G., Bourosh P. (2020). Coordination compounds of copper(II) with Schiff bases based on aromatic carbonyl compounds and hydrazides of carboxylic acids: Synthesis, structures, and properties. Russ. J. Coord. Chem..

[27] Bulhac I., Danilescu O., Rija A., Shova S., Kravtsov V.C., Bourosh P. (2017). Cobalt(II) complexes with pentadentate Schiff bases 2,6-diacetylpyridine hydrazones: Syntheses and structures. Russ. J. Coord. Chem..

[28] Bourosh P., Bulhac I., Mirzac A., Shova S., Danilescu O. (2016). Mono- and dinuclear Vanadium complexes with the pentadentate Schiff base 2,6-diacetylpyridine bis(nicotinylhydrazone): Synthesis and structures. Russ. J. Coord. Chem..

[29] Bellamy L.J. (1957). The Infra-Red Spectra of Complex Molecules.

[30] Gudasi K.B., Patil S.A., Vadavi R.S., Shenoy R.V., Nethaji M., Bligh S.W.A. (2006). Synthesis and spectral investigation of manganese(II), cadmium(II) and oxovanadium(IV) complexes with 2,6-diacetylpyridine bis(2-aminobenzoylhidrazone): Crystal structure of manganese(II), and cadmium(II) complexes. Inorg. Chim. Acta.

[31] Fondo M., Sousa A., Bermejo M.R., Garcia-Deibe A., Sousa-Pedrares A., Hoyos O.L., Helliwell M. (2002). Electrochemical synthesis and X-ray characterisation of cadmium complexes containing 2,6-bis(1-salicyloylhidrazonoethyl)pyridine—The influence of the supporting electrolyte on the nature of the isolated compounds. Eur. J. Inorg. Chem..

[32] Pelizzi C., Pelizzi G., Vitali F. (1987). Investigation into aroylhydrazones as chelating agents. Part 8. Synthesis and spectroscopic characterization of complexes of Co, Ni, Cu, Zn, and Cd with 2,6-diacetylpyridine bis(salicyloylhydrazone); X-ray crystal structure of dichloro[2,6-diacetylpyridine bis(salicyloylhydrazone)]cadmium(II)-chloroform-methanol (1/1/1). J. Chem. Soc. Dalton Trans..

[33] Nithya P., Simpson J., Govindarajan S. (2018). Syntheses, structural diversity and thermal behavior of first row transition metal complexes containing potential multidentate ligands based on 2,6-diacetylpyridine and benzyl carbazate. Polyhedron.

[34] Tyula Y.A., Zabardasti A., Goudarziafshar H., Roudsari M.S., Dusek M., Eigner V. (2017). Template synthesis of two new supramolecular zinc(II) complexes containing pentadentate N_3_O_2_ semicarbazone ligand: Nanostructure synthesis, Hirshfeld surface analysis, and DFT studies. J. Mol. Struct..

[35] Van de Walle A., Tiwary P., de Jong M., Olmsted D.L., Asta M., Dick A., Shin D., Wang Y., Chen L.-Q., Liu Z.-K. (2013). Efficient stochastic generation of special quasirandom structures. Calphad.

[36] Andjelkovic K., Sumar M., Ivanovic-Burmazovic I. (2001). Thermal analysis in structural characterization of hydrazone ligands and their complexes. J. Therm. Anal. Calorim..

[37] Qin Y., She P., Huang X., Huang W., Zhao Q. (2020). Luminescent manganese(II) complexes: Synthesis, properties and optoelectronic applications. Coord. Chem. Rev..

[38] Drzewiecki A., Padlyak B., Adamiv V., Burak Y., Teslyuk I. (2013). EPR spectroscopy of Cu^2+^ and Mn^2+^ in borate glasses. Nukleonika.

[39] Qin B.-W., Zhang X.-Y., Zhang J.-P. (2020). A stable multifunctional cadmium-organic framework based on 2D stacked layers: Effective gas adsorption, and excellent detection of Cr^3+^, CrO_4_^2−^, and Cr_2_O_7_^2−^. Dyes Pigment..

[40] Hua J.-A., Zhao Y., Kang Y.-S., Lu Y., Sun W.-Y. (2015). Solvent-dependent zinc(II) coordination polymers with mixed ligands: Selective sorption and fluorescence sensing. Dalton Trans..

[41] Chisca D., Croitor L., Petuhov O., Kulikova O.V., Volodina G.F., Coropceanu E.B., Masunov A.E., Fonari M.S. (2018). Tuning structures and emissive properties in a series of Zn(II) and Cd(II) coordination polymers containing dicarboxylic acids and nicotinamide pillars. CrystEngComm.

[42] Kravtsov V.C., Lozovan V., Siminel N., Coropceanu E.B., Kulikova O.V., Costriucova N.V., Fonari M.S. (2020). From 1D to 2D Cd(II) and Zn(II) coordination networks by replacing monocarboxylate with dicarboxylates in partnership with azine ligands: Synthesis, crystal structures, inclusion, and emission properties. Molecules.

[43] Mazza P., Zani F., Orcesi M., Pelizzi C., Pelizzi G., Predieri G. (1992). Synthesis, structure, antimicrobial, and genotoxic activities of organotin compounds with 2,6-diacetylpyridine nicotinoyl- and isonicotinoylhydrazones. J. Inorg. Biochem..

[44] Sheldrick G.M. (2007). A short history of SHELX. Acta Crystallogr. A.

[45] Sheldrick G.M. (2015). Crystal structure refinement with SHELXL. Acta Crystallogr. C.

[46] Macrae C.F., Bruno I.J., Chisholm J.A., Edgington P.R., McCabe P., Pidcock E., Rodriguez-Monge L., Taylor R.J., Van De Streek J., Wood P.A. (2008). Mercury CSD 2.0—New features for the visualization and investigation of crystal structures. J. Appl. Crystallogr..

[47] Spek A.L. (2009). Structure validation in chemical crystallography. Acta Crystallogr. D.

[48] Turner M.J., McKinnon J.J., Wolff S.K., Grimwood D.J., Spackman P.R., Jayatilaka D., Spackman M.A. (2017). CrystalExplorer17.

