# Supplementary material for: Crystal Engineering of Schiff Base Zn(II) and Cd(II) Homo- and Zn(II)M(II) (M = Mn or Cd) Heterometallic Coordination Polymers and Their Ability to Accommodate Solvent Guest Molecules
Source: Molecules. 2021 Apr 16;26(8):2317. doi: 10.3390/molecules26082317 (PMC8073138; doi:10.3390/molecules26082317)
Supplement: Supplementary file 1 [file molecules-26-02317-s001.zip › molecules-1175045-supplementary.pdf]

molecules

Supporting information for

Crystal Engineering of Schiff Base Zn(II) and Cd(II) Homo- and Zn(II)M(II) (M = Mn or Cd) Heterometallic Coordination Polymers and Their Ability to Accommodate Solvent Guest Molecules

Olga Danilescu[1], Paulina N. Bourosh[1,2], Oleg Petuhov [1,3], Olga V. Kulikova[2], Ion Bulhac [1], Yurii M. Chumakov[2] and Lilia Croitor[2,*]

[1] Institute of Chemistry, Academy str., 3 MD2028 Chisinau, R. Moldova; olgadanilescu@mail.ru; petuhov.chem@gmail.com, ionbulhac@yahoo.com

[2] Institute of Applied Physics, Academy str. 5, MD2028, Chisinau, R. Moldova; bourosh.xray@phys.asm.md, olga.kulikova@phys.asm.md , xray52@mail.ru, croitor.lilia@gmail.com

[3] Institute of Geology and Seismology, Gheorghe Asachi str., 60/3, Chisinau, R. Moldova; petuhov.chem@gmail.com

* Correspondence: croitor.lilia@gmail.com

molecules

MDPI

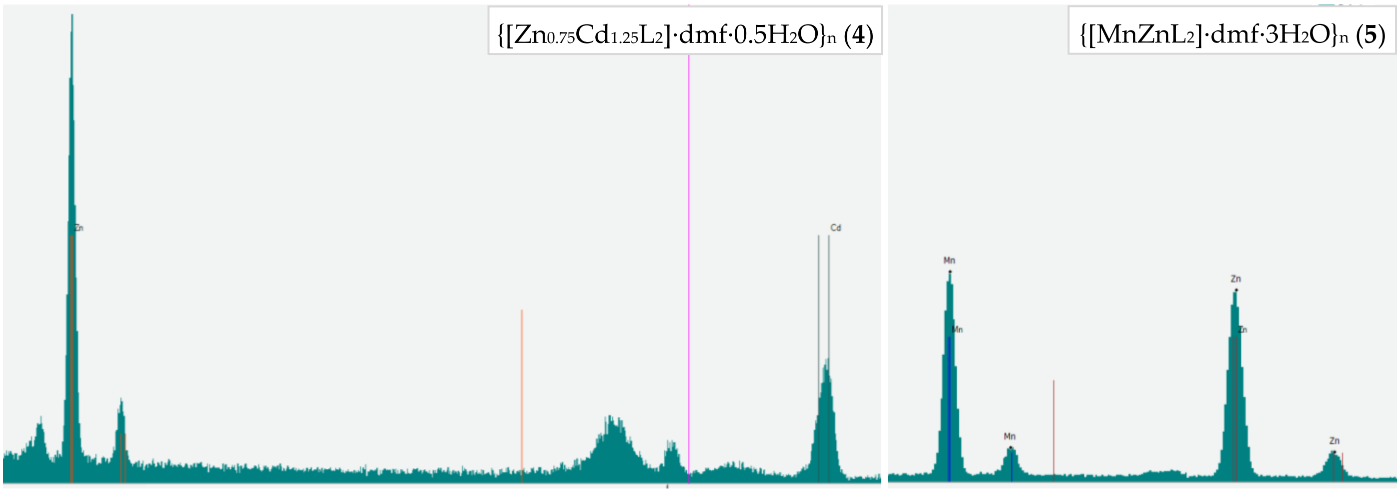

Figure S1. Qualitative report data for compounds **4** and **5**

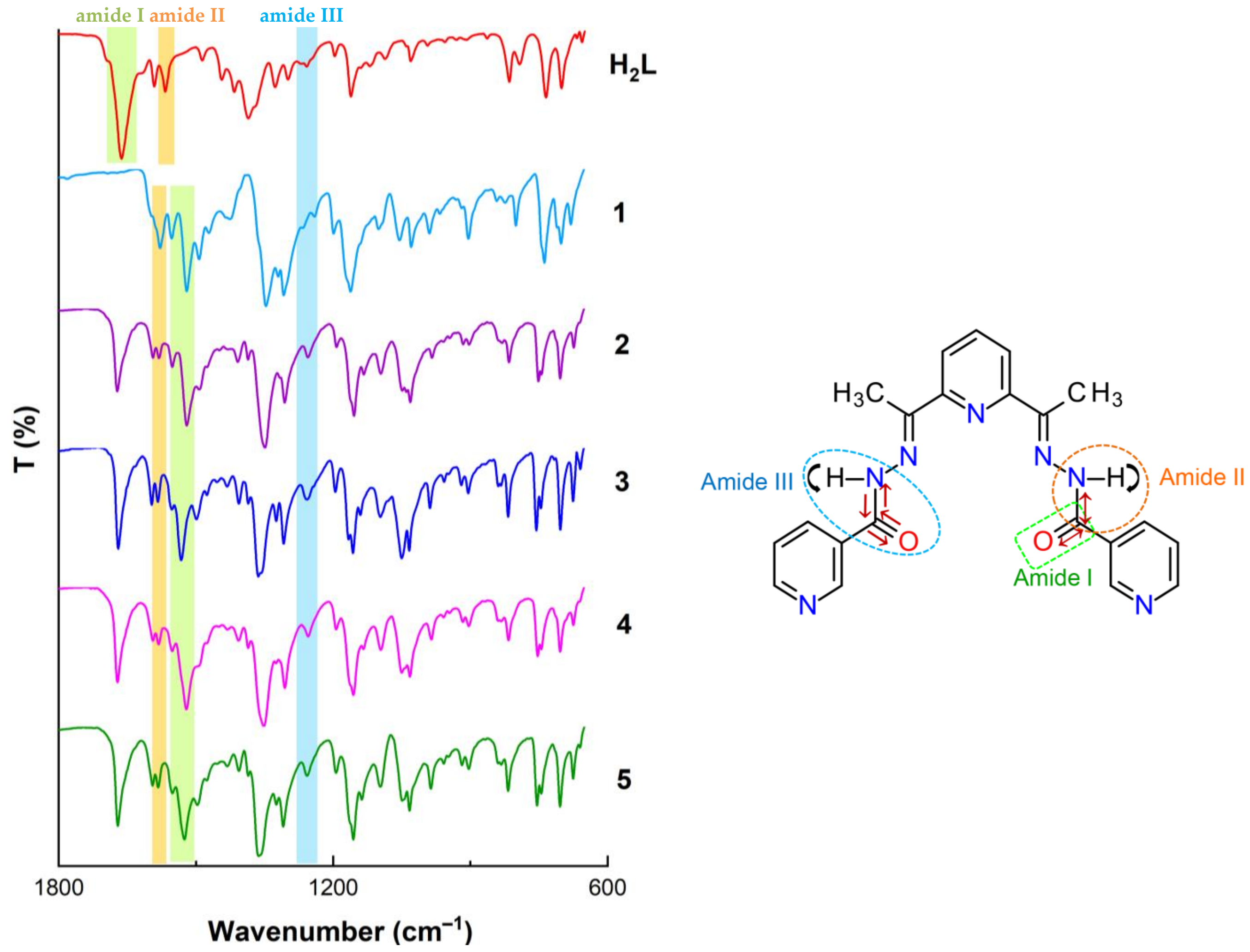

Figure S2. The contribution of Amide vibrational modes in the IR spectra of H_2L and CPs **1-5** in the interval 600–1800 cm^{-1} and their representation on the ligand structure.

molecules

MDPI

Table S1. Selected bond lengths (Å) and angles (°) in coordination metal environment in **1 – 5**.

**Bonds**	**1** (M = Cd), (Å)	**2** (M = Cd), (Å)	**3** (M = Zn), (Å)	**4** (M = Zn/Cd), (Å)	**5** (M = Zn/Mn), (Å)
M(1)–O(1)	2.303(4)	2.323(4)	2.170(3)	2.286(6)	2.190(4)
M(1)–O(2)	2.289(4)	–	–	–	–
M(1)–N(1)#1	2.381(4)	2.419(6)	2.236(4)	2.385(7)	2.286(5)
M(1)–N(3)	2.333(5)	2.368(5)	2.249(4)	2.340(7)	2.263(5)
M(1)–N(4)	2.397(4)	2.402(6)	2.290(5)	2.390(8)	2.310(6)
M(1)–N(5)	2.346(5)	–	–	–	–
**Angles**	(°)	(°)	(°)	(°)	(°)
O(1)–M(1)–O(1)#2/O(2)	87.9(1)	91.2(2)	81.0(2)	89.7(3)	83.0(2)
O(1)–M(1)–N(1)#1	97.0(1)	96.3(2)	92.7(1)	86.3(2)	88.0(2)
O(1)–M(1)–N(3)	68.5(1)	68.2(2)	71.0(1)	68.9(2)	70.5(2)
O(1)–M(1)–N(4)	134.8(1)	134.4(1)	139.5(9)	135.2(2)	138.5(1)
O(1)–M(1)–N(5)	154.5(2)	–	–	–	–
O(1)#2/O(2)–M(1)–N(1)#1	88.4(2)	85.9(2)	88.5(1)	96.0(2)	93.7(2)
O(1)#2/O(2)–M(1)–N(3)	154.6(2)	158.4(2)	151.8(1)	157.8(2)	153.1(2)
O(2)–M(1)–N(4)	134.2(2)	–	–	–	–
O(2)–M(1)–N(5)	68.3(2)	–	–	–	–
N(1)#1–M(1)–N(3)	103.4(2)	90.1(2)	89.0(1)	88.9(3)	90.4(2)
N(1)#1–M(1)–N(4)	99.3(2)	88.5(1)	89.2(1)	88.4(2)	88.9(1)
N(1)#1–M(1)–N(1)#3		177.0(3)	178.5(2)	176.8(4)	177.8(3)
N(1)#1–M(1)–N(3)#2		88.7(2)	90.4(2)	89.8(2)	88.8(2)
N(1)#1–M(1)–N(5)	91.5(2)	–	–	–	–
N(3)–M(1)–N(4)	66.8(2)	66.5(1)	68.5(1)	66.5(2)	68.1(1)
N(3)–M(1)–N(5)	132.5(2)	-	-	–	–
N(3)–M(1)–N(3)#2		133.1(3)	137.1(2)	132.9(3)	136.3(3)
N(4)–M(1)–N(5)	66.5(2)	–	–	–	–
Symmetry transformations used to generate equivalent atoms	#1 $-x+1/2, y-1/2, -z+1/2$	#1 $-x, -y, z$<br>#2 $-x-1/2, y+1/2, -z$<br>#3 $-x+1/2, y-1/2, -z$	#1 $x+1/2, -y+3/2, -z-1$<br>#2 $-x+1, -y+1, z$<br>#3 $-x+1/2, y-1/2, -z-1$	#1 $x+2, -y+2, z$<br>#2 $x+1/2, -y+3/2, -z+2$<br>#3 $-x+3/2, y+1/2, -z+2$	#1 $x-1/2, -y+1/2, -z+1$<br>#2 $-x, -y, z$<br>#3 $-x+1/2, y-1/2, -z+1$

Table S2. Hydrogen bond distances (Å) and angles (°) for compounds **1-5**.

Compound	D–H···A	d(H···A)	d(D···A)	∠(DHA)	Symmetry transformation for acceptor
**1**	C(4)–H(4)···N(5)	2.61	3.309(8)	132	–*x*+1/2, *y*+1/2, –*z*+1/2
	C(5)–H(5)···N(7)	2.68	3.457(11)	141	–*x*+1/2, *y*–1/2, –*z*+1/2
**2**	C(3)–H(3)···O(1)	2.49	3.152(8)	128	–*x*+1/2, *y*+1/2, –*z*
	C(5)–H(5)···O(2)	2.46	3.31(3)	152	–*x*+1/2, *y*–1/2, –*z*–1
	C(5)–H(5)···O(1W)	2.55	3.26(2)	133	*x*+1/2, –*y*+1/2, –*z*–1
**3**	C(3)–H(3)···O(1)	2.39	3.027(6)	126	–*x*+1/2, *y*+1/2, –*z*–1
	C(5)–H(5)···O(2)	2.52	3.37(2)	153	*x*, *y*, *z*–1
	C(5)–H(5)···O(1W)	2.60	3.32(2)	135	*x*, *y*, *z*–1
	O(2W)–H(1)···O(2W)	2.33	2.97(6)	135	–*x*, –*y*+1, *z*
**4**	C(3)–H(3)···O(1)	2.49	3.139(10)	127	*x*–1/2, –*y*+3/2, –*z*+2
	C(5)–H(5)···O(2)	2.42	3.23(2)	146	*x*+1/2, –*y*+1/2, –*z*+2
**5**	C(3)–H(3)···O(1)	2.40	3.044(7)	127	*x*+1/2, –*y*+1/2, –*z*+1
	C(5)–H(5)···O(2)	2.55	3.41(2)	154	–*x*, –*y*+1, *z*+1
	C(5)–H(5)···O(1W)	2.60	3.32(2)	135	*x*, *y*, *z*+1

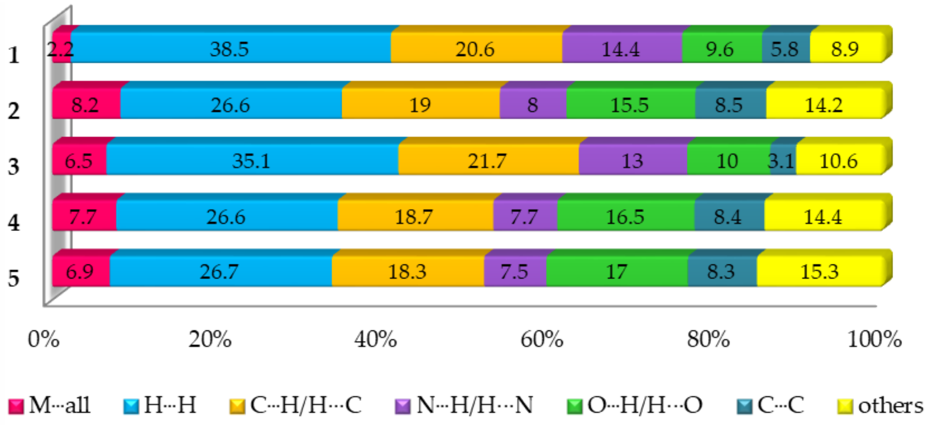

Figure S3. Relative contributions of various intermolecular contacts to the Hirshfeld surface area in compounds **1-5**.

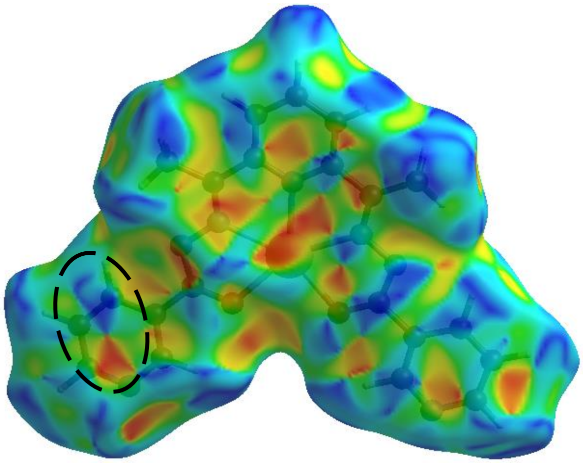

Figure S4. Hirshfeld surface under the shape index function (from -1.0 (red) to 0.995 (blue) Å) demonstrating the presence of stacking interactions in the crystal of compound **1**.

Table S3. Hirshfeld surface properties for compounds **1-5**[a].

Compound	d_{norm} range (Å)	V_H (Å^3)	S_H (Å^2)	G	Ω
**1**	-0.480 – 1.375	487.73	440.08	0.681	0.193
**2**	-1.193 – 1.469	288.53	282.15	0.748	0.302
**3**	-1.191 – 1.365	261.31	265.29	0.745	0.347
**4**	-1.183 – 1.498	284.90	279.27	0.750	0.314
**5**	-1.198 – 1.429	270.89	270.88	0.747	0.335

[a] V_H: surface volume; S_H: Hirshfeld surface area; G: globularity; Ω: asphericity.

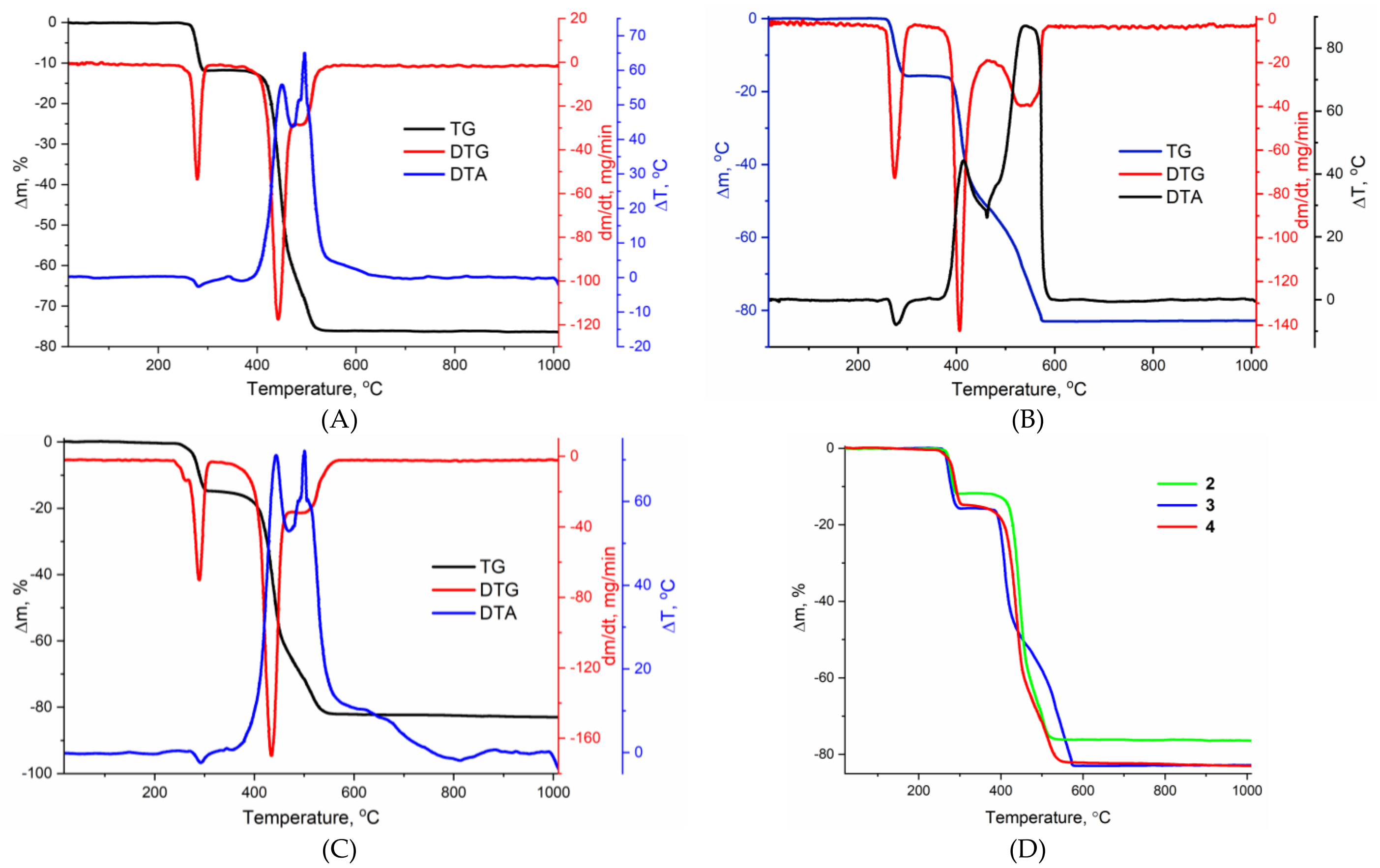

Figure S5. Thermoanalytical curves of compounds **2** - **4** (A-C), as well as comparative TG (D).

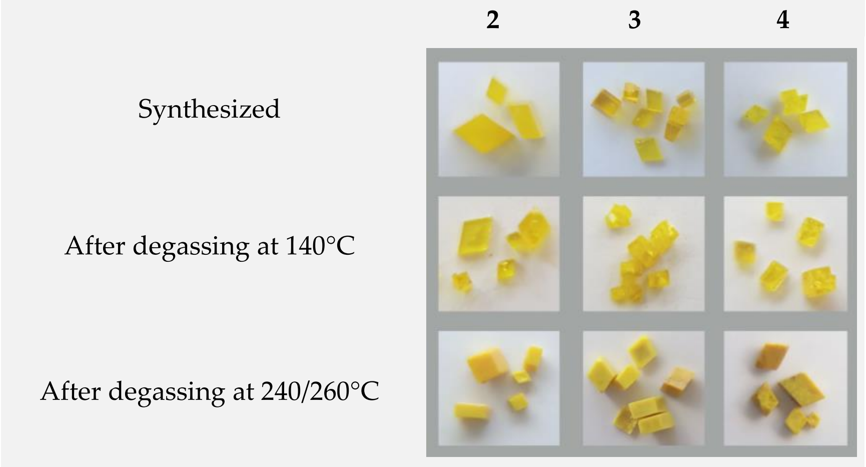

Figure S6. Photos of synthesized **2 - 4** and degassed crystals at various temperatures, demonstrating shape stability and loss of brightness upon removal of guest solvent molecules.

Table S4. Adsorption parameters of studied samples[a].

Compound	**T$_{deg}$, °c**	**S$_{BET}$, m^2/g**	**V$_s$, cm^3/g**
**2**	140	3	0.016
	240	14	0.119
**3**	140	8	0.040
	260	48	0.166
**4**	140	3	0.021
	260	7	0.064

[a] T$_{deg}$: degassing temperature; S$_{BET}$: BET Surface Area; V$_s$: total pore volume

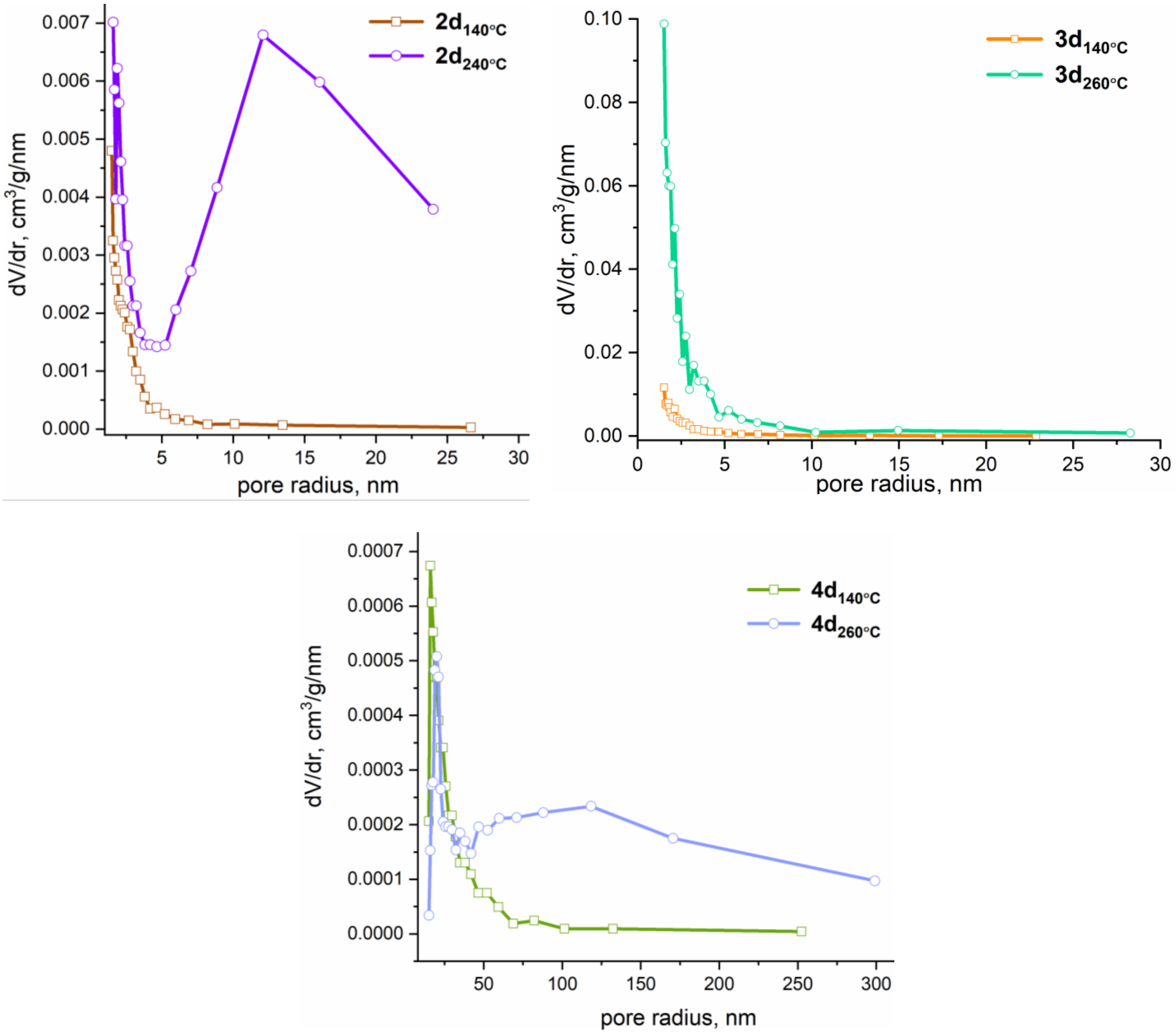

Figure S7. Pore size distribution of degassing samples at different temperatures.
